# Application of Cassia Gum in Enhanced Oil Recovery

**DOI:** 10.1021/acspolymersau.4c00075

**Published:** 2025-02-03

**Authors:** Raíssa Takenaka Rodrigues Carvalho, Neimar Paulo de Freitas, Agatha Densy dos Santos Francisco, Luiz Carlos Palermo, Claudia Regina Elias Mansur

**Affiliations:** † Instituto de Macromoléculas Professora Eloisa Mano, Universidade Federal do Rio de Janeiro, Rio de Janeiro CEP 21945-970, Brazil; ‡ Programa de Engenharia Metalúrgica e de Materiais-PEMM/COPPE, Universidade Federal do Rio de Janeiro, Rio de Janeiro CEP 21941-598, Brazil; § LRAP (Enhanced Oil Recovery Lab), COPPE, Universidade Federal do Rio de Janeiro, Rio de Janeiro CEP 21941-853, Brazil

**Keywords:** Biopolymer, Cassia gum, Enhanced oil recovery, Polymer flooding, Rheology, Sweep efficiency

## Abstract

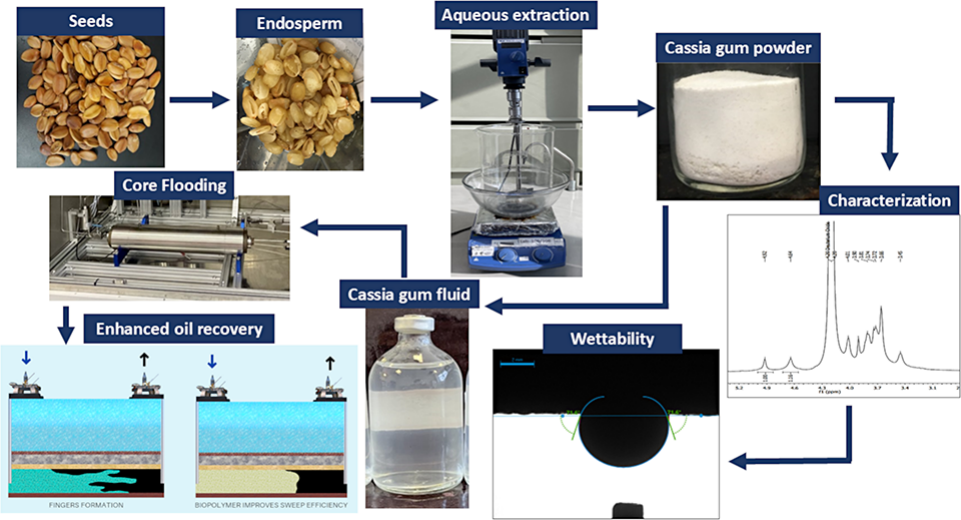

The objective of this study was to develop an innovative biopolymer, cassia gum, for enhanced oil recovery (EOR) applications. The gum was extracted from the seeds of *Cassia grandis*, a native Brazilian tree, using a novel method that achieved an average yield of 24.4 ± 1.7 wt %. Structural characterization identified cassia gum as a nonionic galactomannan with an average molar mass (Mw) of 8.07 × 10^5^ ± 1.44 × 10^5^ g/mol and an organic matter content of 80.32%. A cassia gum-saline solution at 3,000 mg/L, prepared using injection water containing 29,711 mg/L of total dissolved solids, exhibited shear-thinning rheological behavior and viscoelastic properties, with a viscosity of 21.38 cP at 60 °C, closely matching crude oil viscosity. Viscoelastic testing revealed a transition from viscous to elastic behavior, enhancing EOR efficiency by improving sweep and microscopic oil displacement. Contact angle tests with API 25 oil demonstrated that cassia gum could alter carbonate rock wettability from oil-wet to intermediate-wet. Coreflooding experiments under reservoir conditions showed that cassia gum-saline fluid achieved an additional oil recovery of 13.6% OOIP, following 38.5% OOIP recovery during waterflooding. These results establish cassia gum as a promising biopolymer for EOR applications.

## 1 Introduction

In recent years, natural hydrocolloids have been increasingly used in the food industry to improve stabi lity, functional properties, quality and safety, and nutritional and health benefits of different food products such as beverages, bakery and confectionary, sauces and dressings, and meat and poultry In recent years, natural hydrocolloids have been increasingly used in the food industry to improve stabi lity, functional properties, quality and safety, and nutritional and health benefits of different food products such as beverages, bakery and confectionary, sauces and dressings, and meat and poultry In recent years, natural hydrocolloids have been increasingly used in the food industry to improve stabi lity, functional properties, quality and safety, and nutritional and health benefits of different food products such as beverages, bakery and confectionary, sauces and dressings, and meat and poultry In recent years, natural hydrocolloids have been increasingly used in the food industry to improve stabi lity, functional properties, quality and safety, and nutritional and health benefits of different food products such as beverages, bakery and confectionary, sauces and dressings, and meat and poultry In recent years, natural hydrocolloids have been increasingly used in the food industry to improve stabi lity, functional properties, quality and safety, and nutritional and health benefits of different food products such as beverages, bakery and confectionary, sauces and dressings, and meat and poultry uctivity of current hydrocolloid sources and to evaluate differe Polysaccharide biopolymers, sometimes referred to as gums, are biomacromolecules derived from renewable resources. These natural polymers offer several advantages over synthetic polymers, such as biodegradability, eco-friendliness, and compatibility with reservoir conditions. Biopolymers have shown promise in polymer flooding applications due to their viscoelastic properties, which enhance the sweep efficiency of injected fluids and improve oil displacement. A key advantage of biopolymers is observed in their stability in high-salinity environments, as unique helical structures (double or triple helices), rigidity, and uncharged chains are exhibited by them.^1−3^

The use of biopolymers in enhanced oil recovery (EOR) is a growing trend and is expected to soon replace synthetic polymers. This shift aligns with the need for innovations in oil production that are both cost-effective and environmentally friendly.^4^ Injecting high molecular weight polymers is commonly employed to control water–oil mobility in reservoirs by increasing the viscosity of the injection water.^5^ This mobility control reduces the formation of preferential flow paths, or “fingering,″ thereby improving the reservoir’s sweep efficiency and ultimately increasing the oil recovery factor.^6,7^

Partially hydrolyzed polyacrylamide (PHPA) is a commonly used viscosifier in enhanced oil recovery (EOR) fluids, yet its effectiveness is limited in reservoirs with high salinity. Additionally, synthetic polymers, like PHPA, can pose environmental risks due to their toxicity. In contrast, biopolymers, such as polysaccharides, polyesters, or polyamides, are safer for marine life.^2,8,9^ Various biopolymers have been explored for EOR, including carboxymethylcellulose, cellulose, guar gum, hydroxyethylcellulose, lignin, schizophyllan, scleroglucan, wellan gum, and xanthan gum. Specific advantages like shear and thermal stability, compatibility with salts, nontoxicity, and good viscosifying power are offered by these biopolymers. However, drawbacks such as oxidative decomposition, variable properties based on the source, biodegradation, and low filterability are also presented by them.^10^

Ferreira et al.^11^ investigated low-concentration scleroglucan solutions for EOR in offshore carbonate reservoirs, finding that a 500 mg/L concentration delayed water breakthrough (172%), improved oil recovery (159% and 10% at fluid breakthrough and 95% water cut, respectively), and increased ultimate oil recovery by 6.3%, while reducing the water–oil ratio by 38% at 95% water cut. Olabade et al.^12^ developed a biopolymer from potato peel starch hydrolysates, achieving oil recovery rates of 35.72% to 60% in core flood tests across various permeabilities.

Sowunmi et al.^13^ demonstrated that xanthan gum in deionized water achieved a high oil recovery of 63% in sandstone core plugs, outperforming guar gum and arabic gum. Bera et al.^14^ evaluated a 4000 mg/L guar gum solution for EOR, reporting an additional oil recovery of 27.23% OOIP after water flooding, which initially recovered over 45% OOIP, under conditions of 39.57°API crude oil, sandstone core, and 50 °C.

Gbadamosi et al.^15^ investigated okra mucilage, a cellulosic polysaccharide extracted via hot water, for oil displacement in high-temperature and high-pressure conditions. Using carbonate core plugs, the mucilage achieved a 12.7% incremental oil recovery over waterflooding. Buitrago-Rincon et al.^16^ achieved a displacement efficiency of 70.27% OOIP and an incremental oil recovery of 6.6% using xanthan gum in core flooding experiments. Hublik et al.^17^ evaluated xanthan TNCS-ST for EOR, reporting incremental oil recoveries of 10% and 13% in sandstone formations and 38% and 67% in carbonate formations, with the highest recovery observed at 6% NaCl concentration.

Serikov et al.^18^ assessed xanthan gum, welan gum, and potato starch for EOR in limestone cores. Xanthan gum achieved a 30% incremental oil recovery and exhibited prolonged reservoir retention, while welan gum, with a 20% recovery increment, showed strong viscosity retention in saline environments, highlighting its potential.

While each study provides valuable insights into the application of biopolymers for enhanced oil recovery (EOR), further research is essential to optimize their performance under diverse reservoir conditions. This is particularly important for environments involving complex brines, carbonate rocks, elevated temperatures, and crude oil interactions. The current literature lacks sufficient evaluations under these challenging conditions, emphasizing the need for developing and testing new materials tailored to such scenarios.

This study investigated cassia gum as an innovative biopolymer for EOR, highlighting its chemical and structural similarities to guar gum.^19^ Primarily used in pet food for gelling, cassia gum also finds applications in textiles, cosmetics, paper, mining, and water treatment.^20^

Commercial cassia gum is typically derived from *Cassia tora* seeds (*Cassia obtustifolia*),^21,22^ but this study used an innovative source: seeds of *Cassia grandis Linn. f.*, a tree native to the Brazilian Amazon. Rich in galactomannan (75% w/w), its structure features a (1–4)-linked β-D-mannopyranose backbone with α-D-galactose branches. This nonionic polysaccharide is notable for its easy dispersion, exponential viscosity increase with concentration, stability across a wide pH range, and resistance to electrolytes, although it becomes unstable under strongly acidic conditions.^23^

This research aims to evaluate a novel biopolymer source for enhanced oil recovery (EOR) in carbonate reservoirs, distinct from those commonly discussed in the literature. It covers all stages of applying cassia gum, from extraction and characterization to efficiency testing, to validate its potential to increase oil production. Additionally, this study enhances the understanding of the rheological behavior of a biopolymer-based fluid under reservoir conditions, including using brines with high salinity and the typical reservoir temperature (60 °C) of the Brazilian presalt.

## 2 Material and Methods

### 2.1 Material

The seeds of *Cassia grandis* were obtained from the company Arbocenter Ltd.a. (São Paulo, Brazil). The crude oil was from a Brazilian offshore field and had the following properties: °API of 25.5; water content of 1.25%; total acid number (TAN) of 0.481 mg KOH/g; total base number (TBN) of 4.644 mg KOH/g; viscosity of 21.87 cP (at 60 °C and shear rate of 7.37 s^–1^); Saturates 51.0%, Aromatic 24.4%, Resins 21.1%, Asphaltenes 1.9% and Inorganic 1.4%. The carbonate rock was Indiana limestone CB-112, from the Mississippian formation, supplied by Kocurek Industries (Texas, USA), with permeability in the range of 135–220 mD (KCL/N_2_) and porosity of 17–19%. The core used had length of 6” x diameter of 1.5”. Sodium chloride, potassium chloride, calcium chloride dihydrate, other salts and all chemicals used were of analytical grade (ACS reagent, ≥ 99.0%) and obtained from Sigma-Aldrich (USA).

### 2.2 Methods

#### 2.2.1 Cassia Gum Extraction

**Figure 1:**
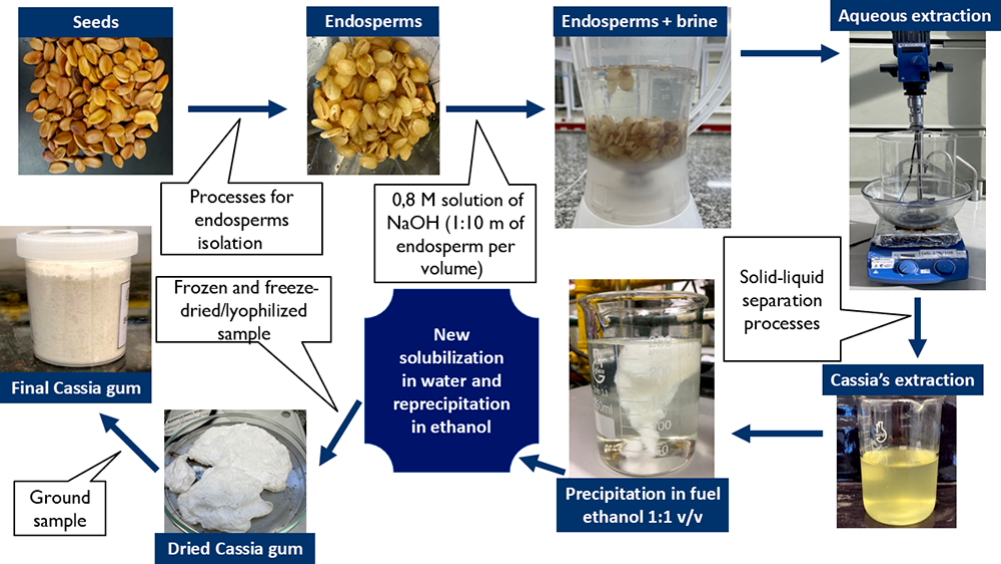
Flowchart of the cassia gum extraction process.

The extraction methodology employed in this study (Figure 1) was adapted from our previous investigation.^24^ In this approach, the seed endosperms were manually isolated, while the seed coats and embryos were discarded. For seeds where endosperms could not be isolated, the samples were dried in an oven at 80 °C until a constant mass was achieved. The difference between the initial and final mass was then used to calculate the extraction yield.

The endosperms were subsequently ground in a blender (Philips Walita -50 s at speed 2) with a 0.8 Molar NaCl solution (1:10 w/v in relation to endosperm mass). The aqueous extraction was conducted under stirring at 500 rpm (IKA RW20 mechanical stirrer) at 42 °C in an oil bath for 8 h. Deionized water was added in the same volume as the brine after 2 and 4 h of extraction.

Following the extraction process, the solution underwent centrifugation (Sigma 3–16P centrifuge with rotor 12155 at 9500 rpm for 10 min). The resulting extract was precipitated in fuel ethanol [1:3 (v/v)] and isolated using a 45 μm standard sieve. The moist gum was then resolubilized in deionized water [1:1 (v/v)] at room temperature^25^ and after this second solubilization, the material was precipitated again in fuel ethanol at 1:1 (v/v) in relation to the new solution. Subsequently, the precipitate was isolated, frozen for 24 h, and lyophilized under vacuum (Liotop K 105 freeze-dryer) at a temperature of −90 °C for 48 h. Finally, the material was ground (IKA A-11 analytical mill) and weighed to determine the final mass (mf).

#### 2.2.2 Characterization of the Cassia Gum

##### Hydrogen Nuclear Magnetic Resonance (^1^H NMR)

The concentration of the cassia gum was 10 mg/mL in deuterated water (deuterium oxide–minimum deuteration degree of 99.9% for NMR spectroscopy, Sigma-Aldrich). The NMR tube measured 7 in. in length and had a diameter of 5 mm, which was inserted in a Varian Mercury VX spectrometer operating at 500 MHz and temperature of 80 °C. The internal standard was sodium 3-(trimethylsilyl)-1-propanesulfonate (DSS).^26^

##### Determination of the Organic Matter Content by Thermogravimetric Analysis (TGA)

Thermogravimetric analysis (TGA) was employed to ascertain the compositions of both organic and inorganic constituents. Approximately 8 mg of gum underwent heating under a nitrogen atmosphere employing, with a heating rate of 10 °C/min, reaching a maximum temperature of 800 °C.^24^

##### Size-Exclusion Chromatography (SEC)

The parameters d*n*/d*c* (refractive index increment), Mw (Weight-Average Molecular Mass), and Mn (Number-Average Molecular Mass) of the cassia gum were determined utilizing an Agilent Technologies 1260 Infinity gel permeation chromatograph equipped with a Shodex LG-G 6B precolumn and two Shodex LB-806 M columns, interfaced with a Wyatt Technology DAWN8+ light scattering detector and Wyatt Technology Optilab T-REX refractive index detector. The mobile phase consisted of 0.1 mol/L NaNO_3_ with 0.025% NaN_3_, and the sample concentration range varied from 0.05 to 2.5 mg/mL. Before analysis, samples were filtered using a 0.22 μm syringe filter. All measurements were conducted at 40 °C with a flow rate of 0.5 mL/min. Data acquisition and analysis were performed using Astra 1.7.3 software.

#### 2.2.3 Rheological Properties

The rheological behavior of cassia gum was evaluated at concentrations of 2,000 and 3,000 mg/L in different synthetic brines at 60 °C. Brine A has a composition that simulates seawater, with Total Dissolved Solids (TDS - discounting water molecules) equal to 29,711 mg/L (NaCl: 27,936.20 mg/L; anhydrous CaCl_2_: 371.63 mg/L; MgCl_2_.6H_2_O: 1,275.03 mg/L; KCl: 748.41 mg/L; Na_2_SO_4_: 57.67 mg/L) and Brine B, which simulates a mixture of 80% injection water and 20% formation water, with a TDS of 68,317 mg/L and a composition described in Carvalho et al.^24^

Rheological assessments were conducted using a TA Instruments DHR 3 rotational rheometer equipped with a 40 mm cone–plate accessory set at a titanium angle of 2° and shear rates ranging from 0.1 to 100 s^–1^.^25^ However, the viscosity value utilized corresponded to a shear rate of 7.37 s^–1^ to simulate the fluid flow within a typical reservoir.^26,27^ Shear stress and shear rate data were fitted with Herschel—Bulkley model.

The viscoelastic properties of the gum were first evaluated using an Oscillation Strain Sweep test with stress ranged from 0.01 to 0.5 Pa at a constant frequency of 1 Hz. This test aimed to identify the gum’s linear viscoelastic region (LVR), enabling the selection of an appropriate stress level for subsequent Frequency Sweep tests. Next, the Frequency Sweep test was conducted over a range of 0.1 to 10 Hz (stress of 0.15 Pa) to evaluate the storage and loss modulus of the fluid.

A similar rheological characterization was performed with crude oil under identical experimental conditions and for the filterability and coreflooding tests, the cassia gum solution was prepared at a concentration of 3,000 mg/L in Brine A. A glutaraldehyde biocide solution (Glutaraldehyde solution, 50 wt % in H_2_O, Sigma-Aldrich) was added, maintaining an equivalent glutaraldehyde concentration (1:1 w/w ratio relative to the gum), and rheological analysis was also conducted at 25 °C.

#### 2.2.4 Filtrability Test

The injectivity of the cassia gum solution (3,000 mg/L of gum +3,000 mg/L of glutaraldehyde) in injection water was evaluated within a system constructed in accordance with API RP 63 (1990), utilizing a stainless-steel filter holder measuring 142 mm (Millipore YY3014236, Merck), under a test pressure of 30 psi at room temperature. A sample volume of 500 mL was initially passed through a Millipore hydrophilic cellulose filter with a pore size of 8 μm, followed by filtration through a filter with a pore size of 1.2 μm. Filtration times (T) were recorded for each test as the filtrate volume reached 20, 40, 60, 80, 120, 140, 160, 180, and 200 mL. Following the methodologies outlined by Levitt and Pope^28^ and Mohd et al.,^29^ the filtration rate (FR) was determined using eq 1:
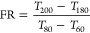
1where *T*_60_, *T*_80_, *T*_180_, and *T*_200_ = Filtration time in seconds when filtrate volume reached 60, 80, 180, 200 mL

#### 2.2.5 Rock–Fluid Interaction and Displacement Tests

##### Rock Wettability (Contact Angle Test)

For the contact angle analysis, slabs were prepared using CB-112 Indiana limestone, with dimensions of 25 mm in length, 15 mm in width, and 5 mm in height, featuring polished surfaces on both faces. Prior to analysis, the slabs underwent cleaning with distilled water (Dubnoff bath, Nova Técnica) under agitation at 200 rpm for 2 days at 60 °C. Following drying, the slabs were subjected to reflux cleaning in a Soxhlet apparatus using toluene, followed by methanol (both solvents for 24 h each). Subsequently, slabs were saturated with formation water for 24 h followed by exposure to crude oil for 30 days at 60 °C.

The contact angle (CA) was quantified utilizing a Drop Shape Analysis (DSA) Hastelloy high-pressure system (Kruss, Hamburg, Germany and Eurotechnica, Bargteheide, Germany), furnished with high-precision pumps, pressure gauges, and temperature regulation capabilities. All experiments were conducted at 60 °C and 1000 psi. The surrounding phases included: solely injection water (system 1); cassia gum in injection water at a concentration of 3,000 mg/L (system 2); a combination of cassia gum and biocide (3,000 mg/L of gum and 3,000 mg/L of glutaraldehyde) (system 3); and the slab postsaturation with oil but prior to contact angle analysis, submerged in a solution of cassia gum and biocide at 3,000 mg/L for 3 days in an oven at 60 °C (system 4).

##### Coreflooding Test

The Indiana limestone core, measuring 6 in. in length and 1.5 in. in diameter, underwent cleaning using the same protocol outlined for the slabs in the previous section. After drying, the core was weighed, and its dimensions were recorded. Porosity and pore volume were then determined using a CoreLab Ultra-Pore 300 porosimeter, employing a confining pressure of 5000 psi and maintaining a temperature of 25 °C within a nitrogen (N_2_) atmosphere. Gas (N_2_) permeability was assessed utilizing a CoreLab Ultra-Perm 610 permeameter.

**Figure 2:**
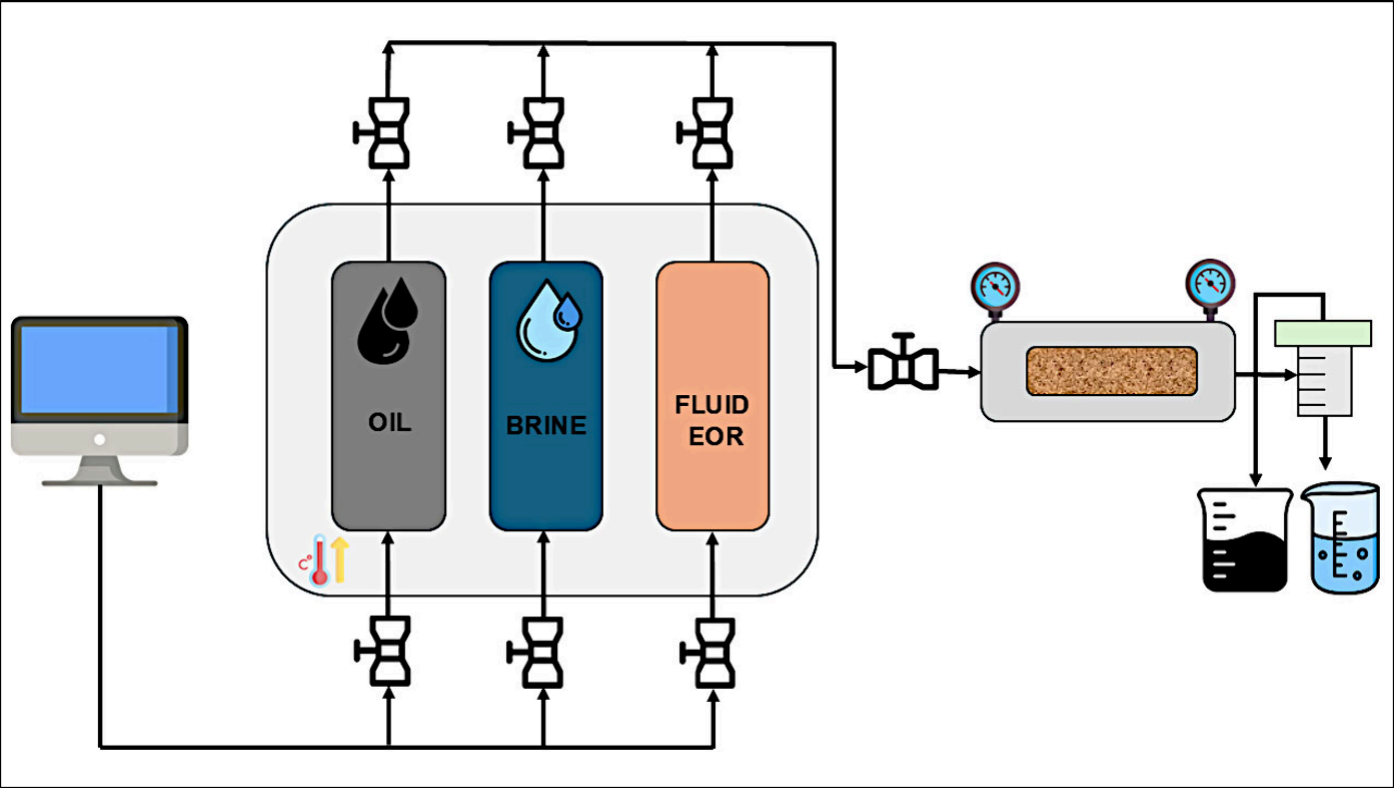
Schematic of the coreflooding apparatus used to evaluate cassia gum.

Following this, the core was saturated with brine in a static saturator under vacuum conditions for 72 h. Following brine saturation, the core was confined at 5000 psi, with a pore pressure of 1000 psi and a temperature of 60 °C (an average for Brazilian Presalt),^30^ while maintaining a constant flow rate of 1.0 cc/min. The equipment utilized is illustrated in Figures 1S and 2S (Supporting Information), with the schematic of the coreflooding apparatus shown in Figure 2.

The procedure commenced with brine injection (TDS: 29,711 mg/L) until pressure stabilization was achieved. Following, crude oil was injected into the core at 0.2; 0.5 and 1 cc/min with injection continuing even after pressure stabilization to allow adequate time to stabilize rock-oil interactions and wettability alteration. Subsequently, secondary recovery (waterflooding) was conducted using synthetic injection water, with the volume of crude oil collected recorded. Postwaterflooding, cassia gum flooding (EOR) was performed to assess additional oil recovery with biopolymer injection. Cassia gum was injected into the core until oil production ceased, and production and differential pressure histories were recorded to calculate the oil recovery factor and residual oil saturation.^31^

## 3 Results and Discussion

### 3.1 Cassia Gum Extraction and Characterization

#### Hydrogen Nuclear Magnetic Resonance (^1^H NMR)

Each monosaccharide unit within a complex carbohydrate structure possesses only one anomeric carbon, typically exhibiting a chemical shift falling within the range of 4 to 6 ppm. Identification of this shift facilitates the determination of the mannose (M) to galactose (G) ratio in galactomannans. This ratio is subject to the influence of various factors, including the plant source, extraction conditions (temperature, duration, and solvents employed), purification method, and climatic variations.^32−34^

**Figure 3:**
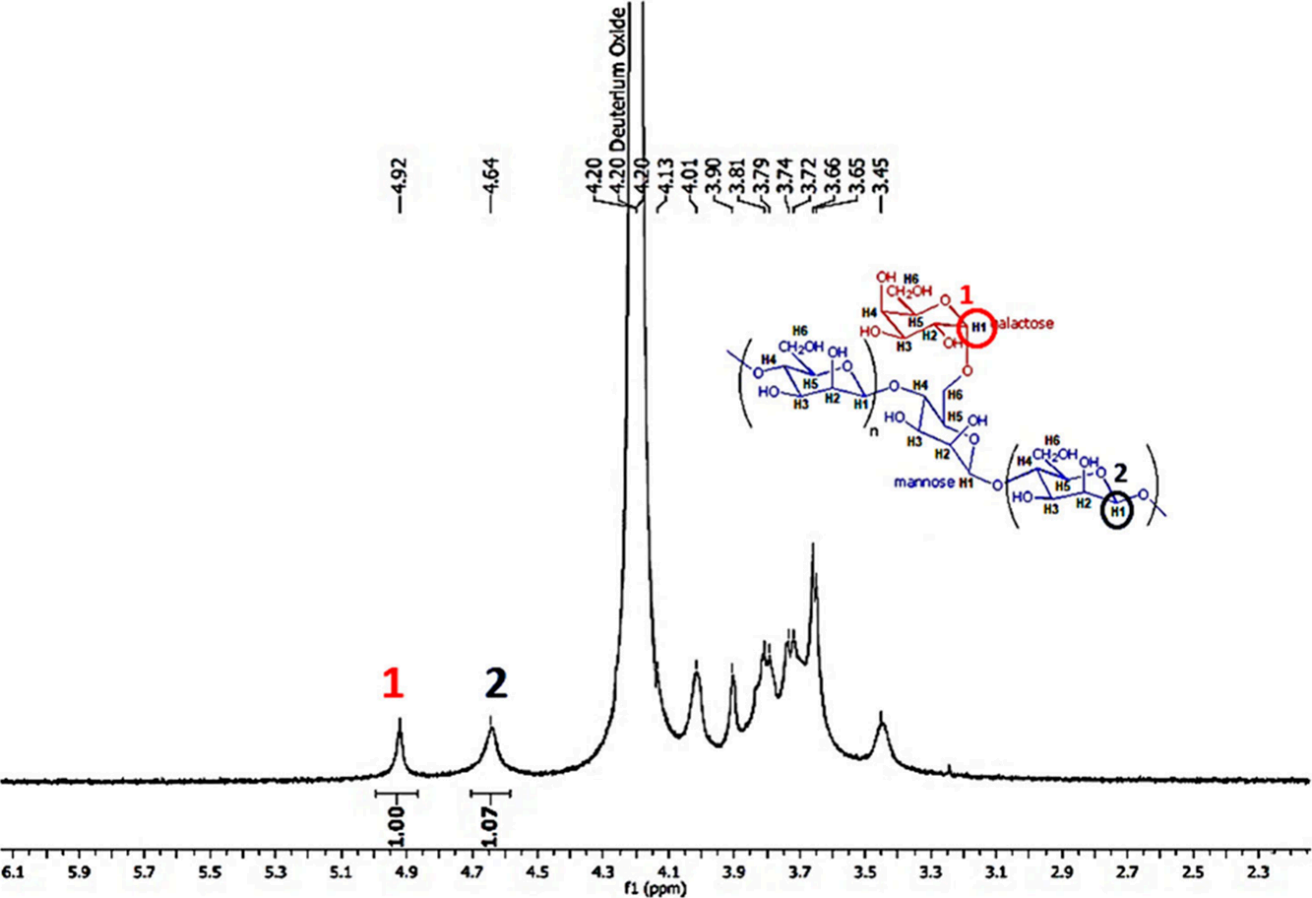
^1^H NMR spectrum of cassia gum extracted from seeds of *Cassia grandis*.

Based on the chemical shifts observed in the NMR spectrum of cassia gum extracted from *Cassia grandis* (Figure 3), the signal at 4.92 ppm was assigned to β-D-galactose, while the signal at 4.64 ppm was assigned to α-D-mannose. The integration of these signals provides a proportional measure of the monosaccharide’s quantity.^35,36^ The M/G ratio determined for the cassia gum was approximately 1, a value consistent with those reported for other galactomannans, such as M/G = 1 for fenugreek gum,^37,38^ 1.39 for guar gum,^39^ and 1.46 for Adenanthera pavonina galactomannan.^35^

#### Determination of the Organic Matter Content by Thermogravimetric Analysis (TGA)

The yield of cassia gum extracted using the novel method outlined in this study from *Cassia grandis* seeds was determined to be 24.63 ± 2.12% m/m. This yield surpassed the values reported by,^24^ where the highest yield achieved was 23.4% w/w. Furthermore, it is emphasized that the yield calculation was based on the initial seed mass (excluding unhulled seeds) and the final biopolymer mass after drying to a constant weight. This yield closely approximated that reported by Jamir et al.^40^ for galactomannan gums extracted from other cassia species. In contrast, Wu et al.^41^ reported a lower yield of 8.02 ± 0.19% m/m for cassia gum extracted from *Cassia obtusifolia* seeds, while Albuquerque et al.^42^ obtained a higher yield of 36 ± 8% w/w for cassia gum, also extracted from *Cassia grandis* seeds. While extraction yield is important, it must be correlated with the gum’s organic matter content, as this influences the viscosity of the injection water and determines the effectiveness of polymer flooding in enhanced oil recovery (EOR) applications.

**Figure 4:**
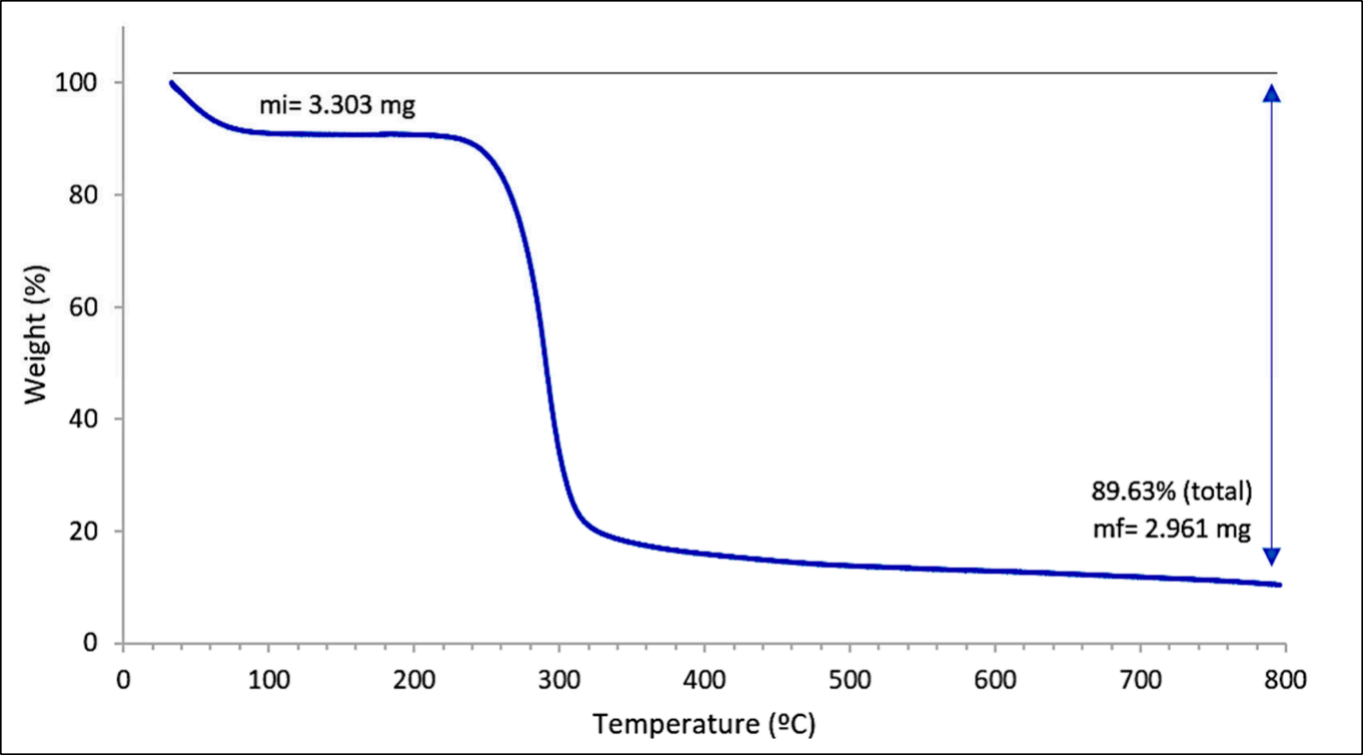
Thermogravimetric analysis curve of cassia gum.

The organic matter content of the cassia gum was determined using Thermogravimetric Analysis (TGA), with the corresponding thermogram depicted in Figure 4. The key events highlighted in the thermogram were identified by Carvalho et al.^24^ The total mass loss recorded was 89.63%. Generally, a higher mass loss indicates a purer cassia gum sample. Subtracting the content of volatiles (9.31% m/m) from this value, the organic matter content was calculated to be 80.32%, with the remaining 10.37% attributed to inorganic matter. It is noted in the literature that a total mass loss approaching 87% is typical for biopolymers derived from natural sources, such as galactomannans.^43,44^

#### Size-Exclusion Chromatography (SEC)

The SEC analyses revealed a d*n*/d*c* value of 0.1652 ± 0.0246 and molar masses of Mw= 8.07 × 10^5^ ± 1.44 × 10^5^ g/mol and Mn= 7.75 × 10^5^ ± 1.39 × 10^5^ g/mol, with a polydispersity index of *M*_w_/*M*_n_ = 1.040 ± 0.004. The calculated weighted average molar mass of the extracted gum exceeded the range reported by Bend et al.,^45^ which specified Mw values between 2–3 × 10^5^ g/mol while Joshi and Kapoor^46^ observed a broader range up to 10^6^ g/mol.

### 3.2 Rheological Properties

**Figure 5:**
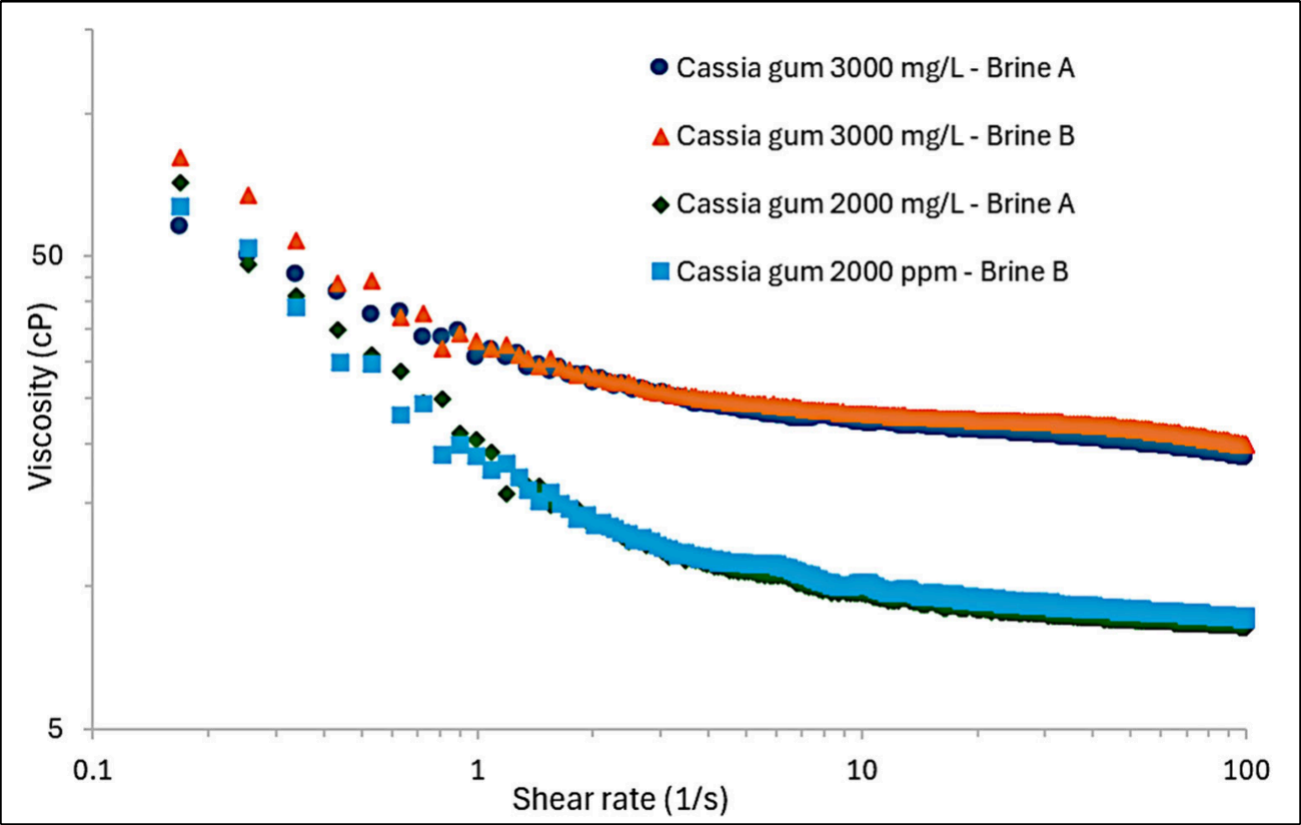
Flow curve of cassia gum with 2000 and 3000 mg/L prepared in injection water (Brine A) and a mixture of 80% injection water and 20% formation water (Brine B) at 60 °C.

In the flow curve shown in Figure 5, the rheological behavior of gum at concentrations of 3,000 mg/L and 2,000 mg/L in Brine A and Brine B at 60 °C depends on the applied shear rate. The data for shear stress versus shear rate were fitted using the Herschel-Bulkley model (rate index: 0.897761) yielding an R^2^ value of 0.9999. As observed in Figure 5, viscosity decreases with increasing shear rate. This behavior occurs due to the entanglement of biopolymer chains, which are abundant at high concentrations and low shear rates. As the shear rate increases, these entanglements are disrupted, leading to the alignment of the chains and, consequently, lower viscosities.^24^ Regarding salinity, it was observed that salinity did not affect the viscosity of the cassia gum solution, as the curves at the same concentration overlapped (Figure 5). This behavior is related to the fact that cassia gum is a nonionic biopolymer. However, the concentration of cassia gum did affect viscosity; higher concentrations led to increased solution viscosity, although this increase was not linear. A concentration of 3,000 mg/L was selected because it demonstrated a viscosity (21.38 cP) similar to crude oil (21.87 cP) under the same conditions.

The nonlinearity of shear viscosity, shear stress, and rate is the result of the viscoelastic fluids ability to store (elastic behavior – *G*′) and dissipate energy (viscous behavior – *G*′’) when the fluid molecule undergoes deformation, such as during the flow through narrow pore channels in reservoirs.^47^Figure 3S (Supporting Information) represents the oscillatory test (0.01 to 0.5 Pa) conducted at a fixed frequency (1 Hz) to identify the linear viscoelastic region, where a predominance of the viscous effect of the fluid is observed, with the viscous modulus being higher than the elastic modulus.

**Figure 6:**
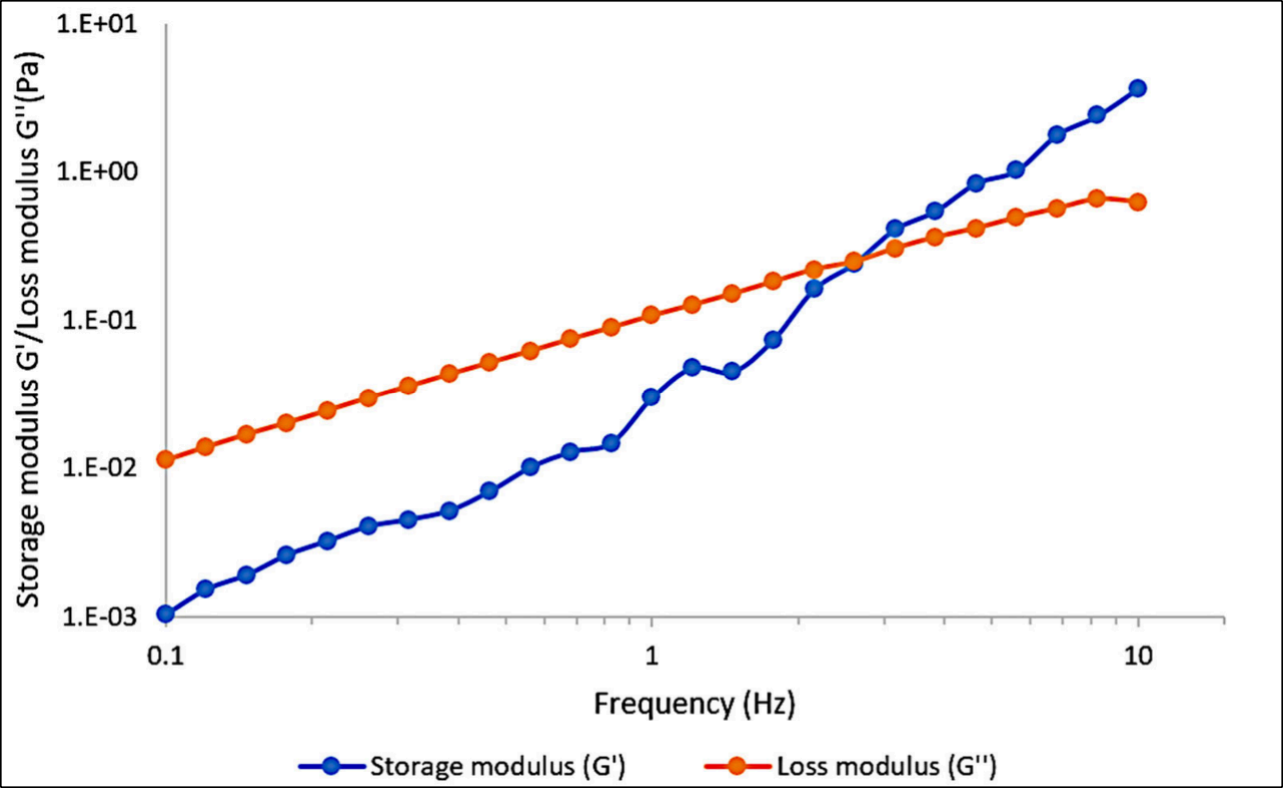
Frequency sweep of cassia gum with 3000 mg/L at 60 °C.

Figure 6 shows *G*′ and *G*″ as a function of frequency within LVR (stress = 0.15 Pa). Cassia gum fluid (3000 mg/L in injection water at 60 °C) is characterized by an evident frequency dependence of the modulus, with a typical liquid behavior at low frequencies (*G*′’ higher than *G*′) and a crossover at higher frequencies. This behavior is typical of dilute polysaccharide water dispersions and depends on the physical entanglement of a chain with a disordered random coil conformation.^48^ The EOR mechanism of viscoelastic polymer flooding is 2-fold. On the one hand, additional viscosity further prohibits viscous fingering so that volumetric sweeping is expanded macroscopically. At the same time, the oil displacement efficiency is enhanced due to the deformation of long-chained molecular structure microscopically so that the residual oil can be hauled out in dead ends or pore throats and on the rock surface.^49^

### 3.3 Filterability Test

The filterability test precedes the displacement test in a porous medium to assess the injectivity and filterability of cassia gum. Table 1S (Supporting Information) presents the filtration times through 8.0 and 1.2 μm membranes for various filtrate volumes from the cassia gum solution.

Using the times outlined in Table 1S and the results from eq 1 (detailed in section 2.2.4), the filterability factors (FR) were determined to be 1.00 for the 8.0 μm membrane and 1.18 for the 1.2 μm membrane. Flaaten^50^ indicates that a filterability factor below 1.20 suggests satisfactory injectivity for a polymer-based fluid. While the factor met this criterion for the 8.0 μm membrane, it approached the threshold for the 1.2 μm membrane.

Aliquots were collected before and after each filtration, and their viscosities were measured at temperatures of 25 and 60 °C. The viscosity values obtained at a shear rate of 7.37 s^–1^ are presented in Table 1.

**Table 1:** Rheology of the Samples from the Filterability Test

System	Viscosity at 7.3 s^–1^ (cP)	Percentage reduction (%)
Cassia gum before filtration →25 °C	53.67	
Cassia gum before filtration →60 °C	21.38	
Cassia gum after 8.0 μm membrane →25 °C	49.44	7.88
Cassia gum after 8.0 μm membrane →60 °C	18.81	12.02
Cassia gum after 1.2 μm membrane →25 °C	45.84	14.59
Cassia gum after 1.2 μm membrane →60 °C	18.26	14.59

As shown in Table 1, the viscosity of the systems decreased after passing through the membranes, indicating polymeric material retention. However, it is suggested that temperature played a predominant role in this retention, as inferred from the test results at 25 °C.

### 3.4 Rock–Fluid Interaction and Displacement Tests

#### Rock Wettability (Contact Angle Test)

**Figure 7:**
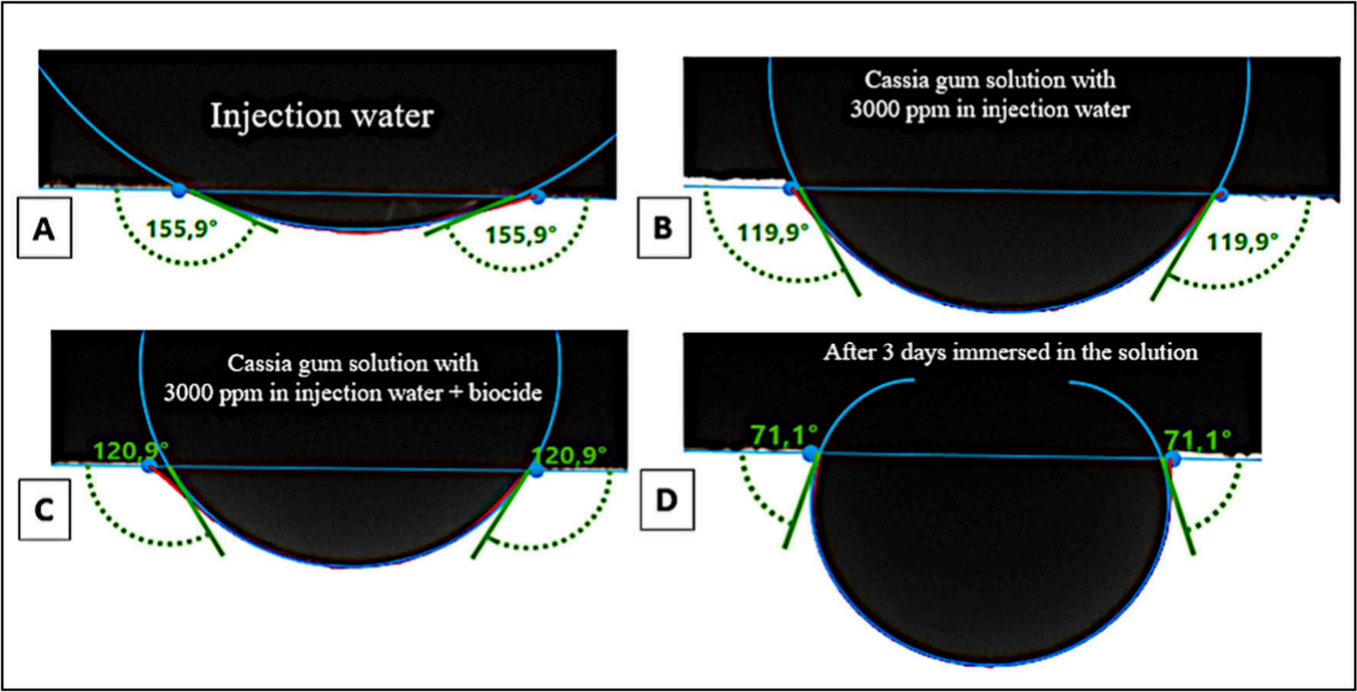
Contact angle analysis with Indiana Limestone and petroleum samples with (a) injection water, (b) gum cassia solution without biocide, (c) gum cassia solution with biocide, and (d) gum cassia solution with biocide after 3 days.

When the surrounding phase was injection water, the contact angle (CA) between the rock and oil droplet was the largest at 155.9°, indicating that carbonate rock (Indiana limestone) exhibited an oil-wet surface after saturation (Figure 7a). This finding aligns with observations made by Høgnesen and colleagues,^51^ who reported that 80–90% of carbonate reservoirs worldwide feature preferentially oil-wet surfaces. The positive surface charge of calcite present in carbonate rocks is attributed as the reason for this preference. Oil wettability in the rock is induced when carboxylates, derived from the carboxylic acids in crude oil, are adsorbed onto the positively charged surface.^52^ The presence of these acids was confirmed by the oil’s Total Acid Number (TAN) value, which measured 0.481 mg KOH/g. Oils with TAN exceeding 0.5 mg KOH/g are classified as acidic oils, indicating that the oil used in this study is close to being acidic. TAN values can also be indicative of reservoir wettability. According to Zhang and colleagues,^53^ reservoirs with TAN values of 0.1 mg KOH/g are preferably water-wet, while those with TAN values of 1 mg KOH/g lean toward oil-wetness. These characteristics are crucial for understanding the behavior of the gum in the reservoir, particularly in relation to fluid–fluid and rock-fluid interactions.

When the surrounding phase was changed to the cassia gum solution with a concentration of 3000 mg/L in injection water, the contact angle (CA) decreased to 119.9° without biocide and 120.9° with biocide (Figure 7b and 7c), thus preserving the previous characteristic of an oil-wet surface with CA > 110°.

In contrast, in the final system analyzed, the contact angle (CA) was notably reduced to 71.1° (Figure 7d), indicating a shift in the rock’s wettability toward the intermediate category. In this system, a slab presaturated with oil was immersed in a cassia gum + biocide solution for 3 days at 60 °C before undergoing contact angle analysis. Some rock-fluid interactions require time to occur, as evidenced in studies on wettability alteration.

During this test, it is hypothesized that a biopolymer film may develop on the surface of the saturated rock, thereby modifying its wettability. In this context, Rellegadla et al.^54^ elucidated that a galactomannan-type biopolymer could be adsorbed onto the rock surface, acting as a dual surfactant layer. The β-linkages in positions 3 and 4 within the mannose or glucose homopolymers facilitate strong and rigid inter-residue hydrogen bonding, which decreases the polymer’s hydration state and enhances the polysaccharide’s hydrophobicity. These hydrophobic residues from the mannose backbone chain act as anchors on the adsorbed oil layer, while the hydroxyl groups of the galactose side chains interact with water molecules, thereby modifying the wettability of the rock.

#### Coreflooding Test

Table 2S in the Supporting Information summarizes the properties of the rock and fluids used in the coreflooding test.

The saturation index of the core under static saturation with synthetic injection water under vacuum was 1. Subsequently, the saturated rock sample underwent a dynamic test, during which the pressure drop (ΔP) stabilized at 1.60 psi, and the absolute permeability to injection water (Kabs) was 196 mDa.

The rock was saturated with oil (0.2; 0.5 and 1 cc/min) following the procedure until the pressure difference stabilized and no water production was observed. A total of 10 pore volumes of oil were injected to achieve core saturation stabilization. The final volume of oil used for saturating the core was measured at 16.9 cc (Table 2), indicating an initial oil saturation (So) of 69.8% and an irreducible water saturation (Swi) of 30.2% (Table 2). The effective permeability of the oil (Ko@Swi) was 161.1 mDa.

**Table 2:** Secondary and Enhanced Oil Recovery with Injection of Cassia Gum

Parameter	Value	Parameter	Value
Absolute water permeability (Kw,abs)	196 mDa	Water mobility	20.9 mD/cP
Initial oil volume (Voi)	**16.9 cc**	Water–oil mobility ratio	2.8^a^
Initial oil saturation (Soi)	69.8%	Cassia gum mobility	13.2 mD/cP
Irreducible water saturation (Swi)	30.2%	Cassia gum-oil mobility ratio	1.8^a^
Volume of oil produced by waterflooding - secondary recovery -(Vor_s)	**6.5 cc**	Volume of oil recovered with biopolymer injection	**2.3 cc**
%OOIPw^a^	38.5%	Total oil recovery (waterflooding + cassia gum flooding)	8.8 cc
Residual saturation oil (Sor)	43.0%	Additional oil recovery with cassia gum	13.6%
Oil permeability (Ko@Swi)	161.1 mD	Total final oil recovery	52.1%
Oil mobility	7.4 mD/cP	Final residual saturation oil	47.9%
Kw@Sor	19.3 mD		

a Dimensionless.

**Figure 8:**
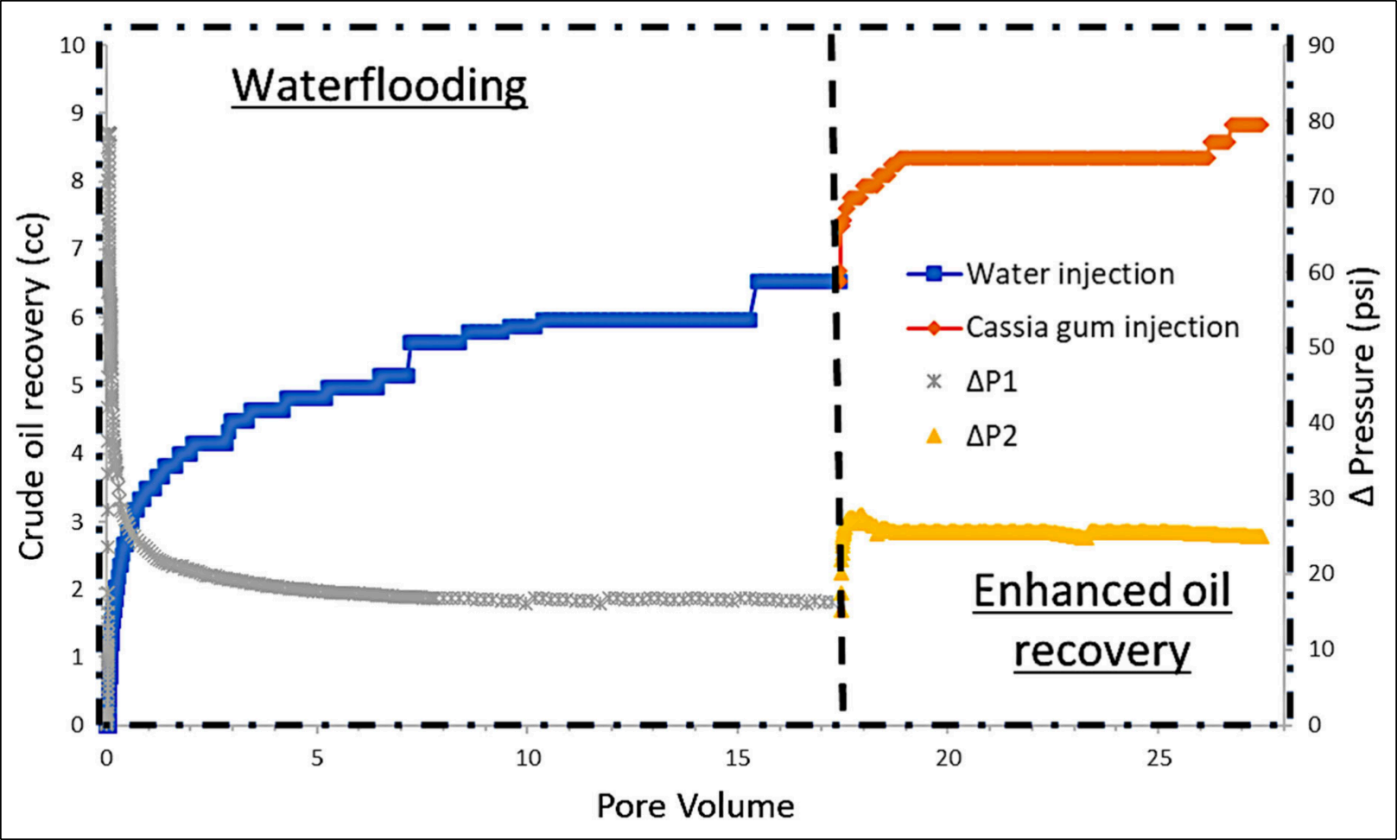
Production of crude oil in secondary and enhanced oil recovery with injection of cassia gum.

After saturating the core with crude oil, brine was injected to simulate the waterflooding recovery process. The breakthrough pore volume, defined as the volume injected until the initial production of water, was measured at 0.34 PV, with oil production nearly stabilizing at 10.0 PV, as shown in Figure 8. However, injection continued up to 17 PV in an attempt to maximize oil recovery. The total volume of oil produced in the waterflooding recovery was 9.4 cc. However, adjusting Figure 8 by deducting the volume attributed to dead oil within the pipeline, totaling 2.9 cc was necessary. Therefore, the final volume of oil produced in this step was 6.5 cc (Table 2), and the original oil in place in waterflooding (%OOIPw) was 38.5% (Figure 8). Comprehensive results are tabulated in Table 2.

The water–oil mobility ratio value, as presented in Table 2, suggests that water exhibited nearly three times greater mobility than oil. This discrepancy likely contributed to the limited oil production observed during the secondary recovery phase, resulting in a recovery of 38.5%OOIP. This finding implies that water may have established preferential flow paths, commonly called “fingers,″ thereby hindering additional oil production.

In previous studies, Feldmann et al.^55^ achieved a secondary recovery efficiency of 35.5% OOIP, Asl et al.^56^ reported 38.6% OOIP, Ghosh et al.^57^ observed 41.0% OOIP, Chaabi et al.^58^ recorded 45.8% OOIP, Olayiwola and Dejam^59^ documented 46.7% OOIP, Moradpour et al.^60^ achieved 52.2% OOIP, and Sari et al.^61^ obtained 53.0% OOIP, all from carbonate rock formations. These relatively low recovery rates can also be attributed to oil’s high wettability of carbonate rocks, as discussed in the preceding section. This characteristic impedes oil displacement by injection water, thereby diminishing secondary recovery efficiency and contributing to the significant volume of residual oil in carbonate reservoirs, ranging from 50 to 68%,^52^ surpassing the value observed in this study of 42.9%.

Therefore, it is apparent that a substantial fraction of the oil persisted within the core. Consequently, the cassia gum solution, derived from the filterability test, was injected until both ΔP and oil production stabilized, resulting in a production of 2.3 cc. This additional production led to a commendable increase in oil recovery, amounting to 13.6% OOIP (see Figure 8). The injection of cassia gum solution caused an increase in the viscosity of the displacing fluid, which reduced the difference in the phase mobility (biopolymer solution and oil), leading to a low mobility ratio. Reducing the mobility ratio improves the sweeping efficiency and increases the oil recovery factor.^62^ With the 13.6% increase in oil recovery using cassia gum, the final total oil recovery reached 52.1%OOIP.

Alzahid et al.^63^ injected xanthan gum biopolymer, selected due to its initial compatibility with brine samples and its comparatively lower rates of filtration and adsorption when juxtaposed with four other biopolymers. A supplementary oil recovery of 5.2% was achieved using a carbonate rock sample through the injection of the biopolymer. An initial oil recovery rate of 74.0% was achieved with water injection by Musa et al.,^64^ and recovery was subsequently enhanced by an additional 16.0% through the injection of 5000 mg/L of guar gum.

Filho and Moreno^65^ utilized a guar gum concentration of 2500 mg/L, resulting in a 13.0% increase in oil recovery. Concerning nonionic biopolymers, Liang et al.,^66^ Castro et al.,^67^ and Ferreira and Moreno^11^ investigated the biopolymer scleroglucan at concentrations of 2500 mg/L, 935 mg/L and 500 mg/L, respectively, achieving additional oil recoveries of 13.0%, 18.0%, and 6.3%. Compared to these results, our findings with cassia gum demonstrate excellent performance, approaching the average reported for other biopolymers.

## 4 Conclusions

The study successfully demonstrated the extraction of cassia gum from an unconventional source, the seeds of the *Cassia grandis* tree, for use in enhanced oil recovery (EOR), achieving promising results. Characterization of the cassia gum using ^1^H NMR confirmed its chemical structure as primarily galactomannan, a nonionic polysaccharide composed of mannose and galactose in a 1:1 ratio, with an average molecular weight of 8.07 × 10^5^ g/mol. Thermal degradation analysis (TGA) revealed an organic matter content of 80.32% m/m, validating the effectiveness of the extraction method developed.

Cassia gum solutions exhibit rheological and viscoelastic properties ideal for enhanced oil recovery (EOR). At concentrations of 3,000 mg/L and 2,000 mg/L in brines at 60 °C, the solutions display shear-thinning behavior, with viscosity decreasing as shear rate increases due to the disruption of biopolymer chain entanglements. Salinity does not affect viscosity, owing to cassia gum’s nonionic nature, but higher concentrations increase viscosity, with 3,000 mg/L identified as optimal for EOR, closely matching the viscosity of crude oil.

Viscoelastic tests revealed a predominantly viscous behavior at low frequencies and a transition to elastic behavior at higher frequencies, characteristic of polysaccharide dispersions with chain entanglements. This dual behavior enhances EOR through increased sweep efficiency and improved microscopic oil displacement by mobilizing residual oil in pores and on rock surfaces.

Rock-fluid interaction tests showed that while the rock surface was initially oil-wet, the introduction of a saline solution containing cassia gum and biocide altered the wettability to an intermediate state over time. This change is favorable for EOR applications.

Coreflooding tests further confirmed the effectiveness of the cassia gum-based fluid. Initial brine injection recovered 38.5% OOIP. The subsequent injection of the saline cassia gum solution, due to its higher viscosity, led to an additional recovery of 13.6%, resulting in a total oil recovery of 52.1% OOIP.

The substantial additional oil recovery achieved highlights the potential of cassia gum as a viable EOR agent. Despite challenges in quantifying the broader advantages of this method, the results suggest that the nonionic biopolymer derived from a novel source can be considered a promising candidate for EOR applications, offering an effective and sustainable alternative for enhanced oil production.

## Supporting Information Available

Internal and external photos of the equipment used in the coreflooding test, a stress sweep graph of cassia gum, and tables with additional information on the filterability tests and the coreflooding test (PDF)
