# Supplementary material for: Application of Cassia Gum in Enhanced Oil Recovery
Source: ACS Polym Au. 2025 Feb 3;5(2):134–44. doi: 10.1021/acspolymersau.4c00075 (PMC11986721; doi:10.1021/acspolymersau.4c00075)
Supplement: Supplementary file 1 — lg4c00075_si_001.pdf [file lg4c00075_si_001.pdf]

SUPPORTING INFORMATION

APPLICATION OF CASSIA GUM IN ENHANCED OIL RECOVERY

Raíssa Takenaka Rodrigues Carvalho [a] , Neimar Paulo de Freitas [a], Agatha Densy dos Santos Francisco[c], Luiz Carlos Palermo[a] and Claudia Regina Elias Mansur *[a,b]

[a]Instituto de Macromoléculas Professora Eloisa Mano, Universidade Federal do Rio de Janeiro, Rio de Janeiro, CEP 21945-970, Brazil

[b]Programa de Engenharia Metalúrgica e de Materiais-PEMM/COPPE, Universidade Federal do Rio de Janeiro, CEP 21941-598, Brazil

[c] LRAP (Enhanced Oil Recovery Lab), COPPE, Universidade Federal do Rio de Janeiro, Rio de Janeiro, CEP 21941-853, Brazil

* ***celias@ima.ufrj.br***

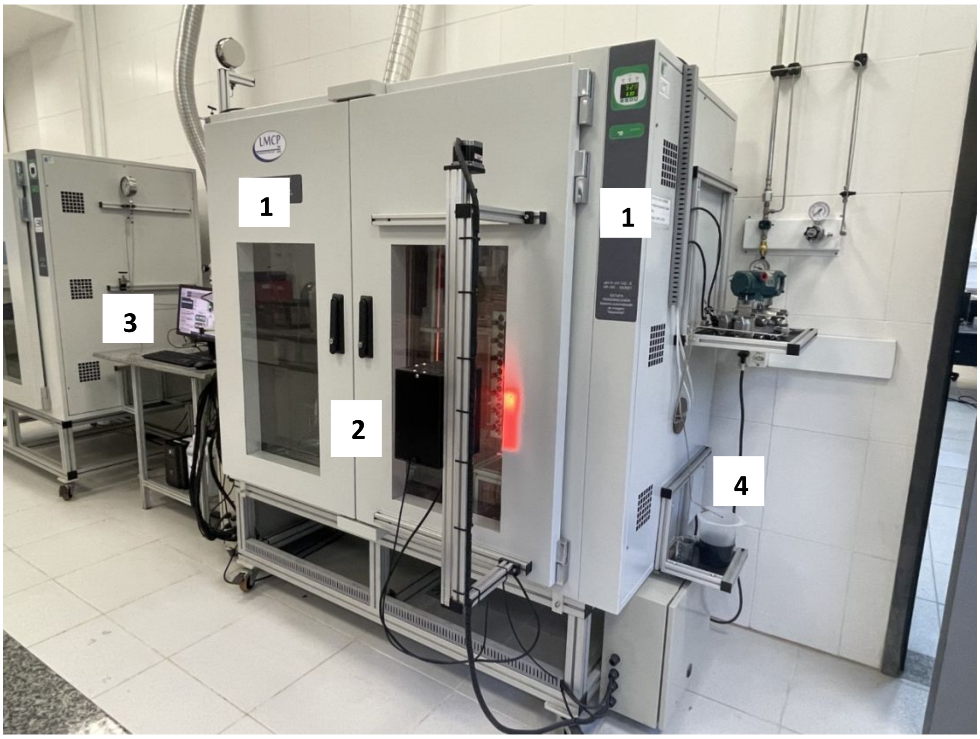

Figure 1S. Equipment used for elution test in porous media (coreflooding). (1) Customized laboratory oven, (2) Camera system for monitoring the volume produced, (3) Computer with customized interface (4) Output and collection of the fluid produced.

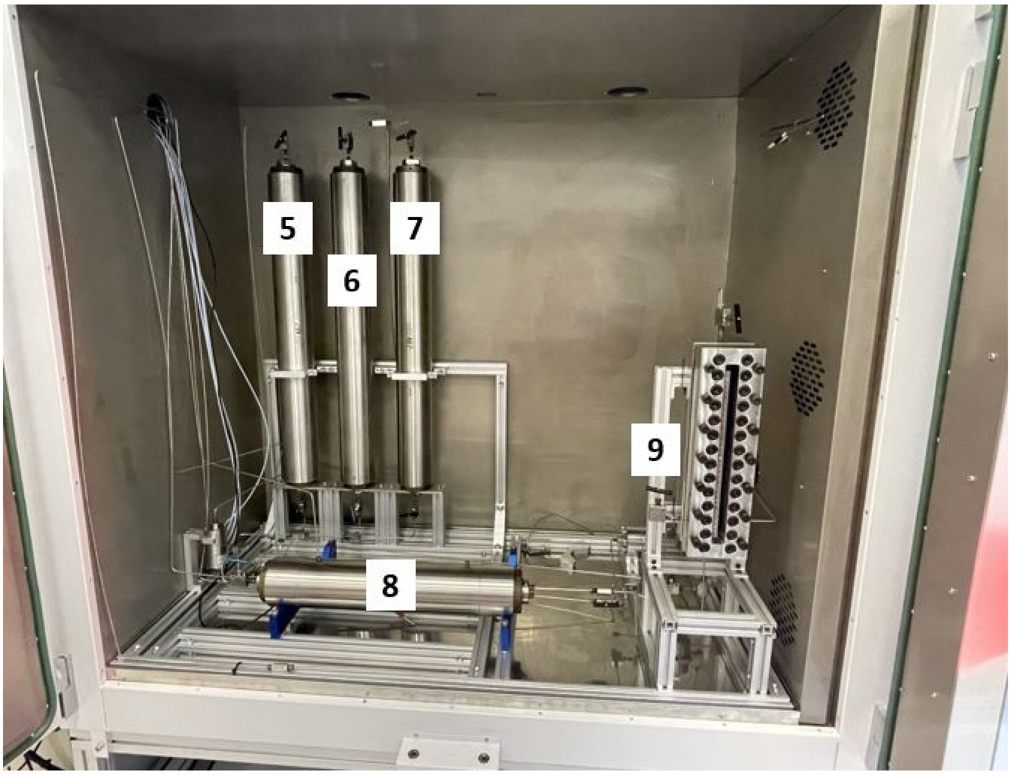

Figure 2S. Laboratory oven internal part that makes up the elution equipment in porous media (coreflooding). (5-7) Displacement bottles for fluid storage (brine, petroleum and biopolymer solution), (8) Coreholder for core confinement and (9) Two-phase phase separator.

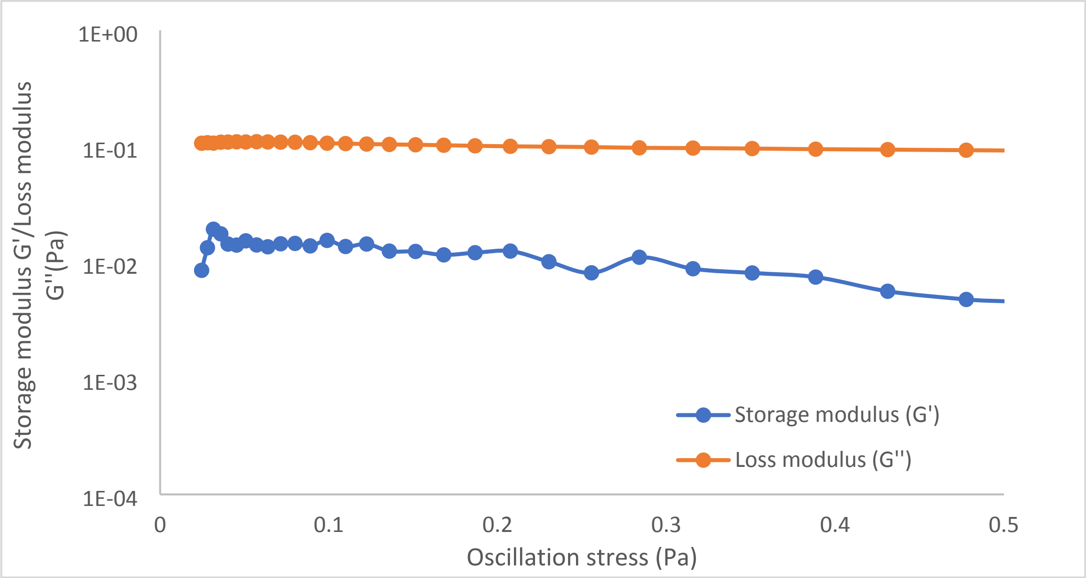

Figure 3S. Stress sweep at 60ºC of cassia gum with 3000 mg/L.

Tabela 1S. Filterability test with cassia gum solution.

	Membrane of 8 µm	Membrane of 1.2 µm
Filtrate volume (mL)	Time (s)	
20	14	13
40	28	30
60	41	50
80	57	72
100	71	89
120	85	109
140	100	129
160	116	152
180	132	172
200	148	198
220	165	220

Tabela 2S. Properties of the core and fluids used in coreflooding test.

**CORE PROPERTIES**		
Parameter	Quantity	Unit
Dry weight	382.1	g
Length	15.3	inch
Diameter	3.76	inch
Face area	11.1	cm^2
Pore volume	24.2	cc
Porosity	14.9	%
Gas permeability	215.7	mD
**PROPERTIES OF FLUIDS (60ºC)**		
Injection water viscosity	0.92	cP
Oil viscosity	21.87	cP
Viscosity of cassia gum + biocide solution	18.26	cP
